# Effect of mixed hay supplementation during fattening on carcass traits and meat quality of Hanwoo steers

**DOI:** 10.1186/s40781-017-0131-y

**Published:** 2017-03-13

**Authors:** Dicky Tri Utama, Ji Hye Choi, Chang Woo Lee, Yeon Soo Park, Sung Ki Lee

**Affiliations:** 1 College of Animal Life Sciences, Graduate School, Kangwon National University, Chuncheon, 24341 South Korea; 2 Gangwon Provincial Livestock Research Center, Hoengseong, 25266 South Korea; 3 Animal Products and Food Science Program, Division of Animal Applied Science, College of Animal Life Sciences, Kangwon National University, Chuncheon, 24341 South Korea

**Keywords:** Beef, Hanwoo, Hay, Meat quality, Orchard grass, Red clover, Straw, Tall fescue, Timothy grass

## Abstract

**Background:**

This study was aim to observe the effects of feeding mixed local hay (MH) consisted of 55% orchard grass (*Dactylis glomerata* L.), 35% tall fescue (*Festuca arundinacea*) and 10% red clover (*Trifolium pratense*) to Hanwoo steers on performance, carcass characteristics and meat quality (*longissimus thoracis*) compared with feeding imported timothy hay (TH) and local rice straw (RS).

**Results:**

Although no significant effects were found on animal performance and carcass yield grade, the carcasses of MH group had higher marbling score and quality grade than those of RS and TH group (*P* < 0.05). Therefore, higher fat content (*P* < 0.001), lower shear force and hardness value in the beef of MH group than that of other groups were observed. Furthermore, the beef of MH group had higher CIE a* value (redness) than that of other groups and feeding MH to Hanwoo steers lowered n-6 to n-3 fatty acids ratio in beef.

**Conclusions:**

Mixed hay provided benefits on meat quality and could be used for Hanwoo fattening program.

## Background

The rapid economic change in Korea leads to the increasing demand of high quality beef. Hanwoo is a major beef cattle raised for highly marbled beef production in Korea, accounted about 85% of slaughtered cattle in 2016 [1]. As the demand of highly marbled beef increases, the use of grain-based diet during fattening also increases among beef cattle industries. However, acidosis becomes a problem when beef cattle entering fattening program due to excess supply of fermentable carbohydrate from concentrate feeding [2]. The inclusion of roughage in diet during fattening program has objective to control acidosis with impact to maintain the integrity and health of ruminal papillae and normal rates of nutrient absorption [3]. Furthermore, the higher content of n-6 fatty acids in grain-fed beef provides unbalance n-6 to n-3 essential fatty acids ratio in human diet that may lead to the occurrence of cardiovascular diseases [4]. Grass-based feeding is well known to reduce this risk by increasing the amount of n-3 essential fatty acids in beef [5]. Thus, adding roughage, hay in particular, could contribute to the health status of beef cattle and the production of healthy meat.

Most of hay products used as feed for beef cattle fattening program in Korea are produced locally. However, the quality remains lower than that of imported hay product as the main local product is rice straw. In 2011, rice straw accounted for 51.40% of total hay product produced locally and about 17.40% of the hay product was imported. Korean feed company utilized 80% of imported hay for total mixed ration (TMR) production, while the rest was distributed as roughage [6]. Van Soest [7] mentioned that rice straw contains high silica that contributes to its lower digestibility in ruminant. Although ammonia or urea treatment can enhance the intake and digestibility of rice straw, lower acceptance was reported as the concern of costs, labor, equipment, health, environment and social issues. Therefore, Korean government promotes local production of high quality hay product through mixing some grasses and legumes that are available from local source. The objective of this study was to compare the effects between mixed hay, rice straw and imported timothy hay supplementation during fattening on carcass traits and meat quality of Hanwoo steers.

## Methods

### Animals and diets

A total of 18 Hanwoo steers (25 ± 1.00 months old, 628 ± 61.84 kg) were randomly allocated to group pen (5 × 10 m) and fed for 150 days (May to October) at Gangwon Provincial Livestock Research Center’s farm. Each pen consisted of six animals with one diet treatment. Three different supplementary hay were used as treatment, as follows; rice straw as control (RS), mixed hay (MH) consisted of 55% orchard grass (*Dactylis glomerata* L.), 35% tall fescue (*Festuca arundinacea*) and 10% red clover (*Trifolium pratense*), which were grown and harvested from local region and imported timothy hay (TH). Nutrient composition of supplementary hay is shown in Table 1. Hay was given *ad libitum* three times a day (7:30, 13:00 and 18:00) following concentrate (12% of crude protein and 73% of total digestible nutrient). Given feed and residues were weighed and recorded daily. The animals had free access to water. Proximate composition, calcium and phosphor content of the forage were determined by the Association of Analytical Communities (AOAC) official methods [8] and detergent analysis was used for determining acid detergent and neutral detergent fiber [9].

**Table 1:** Chemical composition of supplementary hay

	RS	MH	TH
Chemical composition			
Dry matter (% as fed)	89.40	89.60	88.73
Crude protein (% DM)	6.02	7.43	9.41
Crude fat (% DM)	1.72	2.66	2.20
Crude fiber (% DM)	32.02	37.43	36.55
Ash (% DM)	8.32	7.73	6.39
Calcium (% DM)	1.24	1.22	1.17
Phosphorus (% DM)	0.63	0.52	0.65
Acid detergent fiber (% DM)	39.77	32.44	33.23
Neutral detergent fiber (% DM)	55.47	64.03	63.11

*RS* rice straw, *MH* mixed hay, *TH* timothy hay

### Carcass traits evaluation

After five months feeding period, the animals were slaughtered at local abattoir (Hoengseong, Korea) using standard procedure. The carcasses were weighed and then chilled at 2 ± 2 °C for 24 h in chilling room. Carcass yield and grade were estimated using the Korean carcass grading system [10]. Parameters used to determine yield grade (hot carcass weight, backfat thickness and ribeye area) and quality grade (marbling, meat color, fat color, firmness and maturity) were recorded. The *longissimus thoracis* muscle (rib eye) of each animal was collected from the left carcass for meat quality analysis. Samples were packed in polyethylene zipper bags, transported to laboratory and stored at −24 °C until analysis.

### Meat quality analysis

#### Proximate composition analysis

The frozen samples were thawed at 2 ± 2 °C in chilling room for 36 h until internal temperature of 3 to 5 °C was reached. Sample was ground using a food blender at minimum speed for 10 sec (HMF-1600 PB, Hanil Electric, South Korea). Proximate composition was determined by AOAC official methods [8]. Moisture content was determined by drying the samples in the oven at 105 °C for 24 h. Crude fat content was determined by ether extraction using Soxhlet system. Nitrogen content was determined using the Kjeltec system (2200 Kjeltec Auto Distillation Unit, Foss, Sweden) and crude protein was calculated as nitrogen content multiplied by 6.25. Crude ash was determined by burning the samples in the muffle furnace at 550 °C for 12 h.

#### Instrumental surface color

The surface color was recorded by measuring the Commission International De L’ecairage (CIE) lightness (L*), redness (a*) and yellowness (b*) using a chromameter (CR-400, Konica Minolta Sensing Inc., Japan). The light source of illuminant C (2° observer) was standardized with a white plate (Y = 93.6, X = 0.3134, y = 0.3194). Surface color was assessed in triplicate.

#### pH, water holding capacity and drip loss

For pH measurement, a total of 5 g sample was added with 45 mL of distilled water and then homogenized at 10,000 rpm for 60 s using a homogenizer (PH91, SMT Co., Ltd., Japan). The pH value of the homogenized samples were recorded using a pH meter (SevenEasy pH, Mettler-Toledo GmbH, Switzerland). Water holding capacity (WHC) was defined as the percentage of moisture content that was hold by the meat during heating with centrifugation method [11]. Briefly, 5 g of ground samples were weighed into graduated centrifuge tubes, sealed and heated for 30 min in a 75 °C water bath. The tubes were cooled in flowing water for another 30 min, centrifuged at 980 g for 10 min at 24 °C. The supernatant was decanted and measured, and the moisture content of both raw sample and the supernatant was determined by AOAC method [8]. For drip loss, the samples were cut (1.5 cm thick), packed in polyethylene zipper bags and stored at 2 ± 1 °C for 6 days and then weighed. Drip loss percentage was expressed as weight loss during storage as described by Honikel [12].

#### Cooking loss and textural properties

The 2.5 cm thick-samples (70 g) were cooked in the polyethylene zipper bags until internal temperature of 72 °C was reached by immersing in water bath. The cooked samples were then removed off the polyethylene bags immediately, cooled down and weighed. Cooking loss was expressed as the percentage of weight loss during boiling. The cooked samples were cut (1 × 1 × 1 cm) and subjected to texture profile analysis and shear force measurement using TA-XT2i Plus (Stable Micro Systems, UK). For shear force measurement, the cut sample was placed on the table, under the V blade, and was cut through as the blade moved down with a constant speed through the slit of the table (assay parameters were: pre-test speed: 2.0 mm/s; test speed: 1.0 mm/s; post-test speed: 5.0 mm/s). A cylindrical 35 mm-diameter probe was used for all texture profile analysis in this study. The sample was placed under the probe that moved downwards at a constant speed of 2.0 mm/s (pre-test), 1.0 m/s (test), and 5.0 mm/s (post-test). Each assay was conducted using eight cuts from each sample.

### Fatty acid composition

Fatty acid composition was determined using a gas chromatography (YL6500, YL Instrument, Korea). Lipid was extracted according to Folch et al. [13] with chloroform-methanol (2:1 v/v). Each sample was assessed 2 times. Fatty acids were converted into methyl esters as described by AOAC [8] with modifications. Briefly, 200 μL of lipid sample and 2 mL of 2 N NaOH were vortex-mixed and heated at 80 °C for 60 min. The samples were cooled by immersing in cold water, vortex-mixed with 2 mL of 25% BF_3_-MeOH, heated again at 80 °C for 60 min. After cooling for 10 min in cold water, the samples were mixed with 3 mL of distilled water and 2 mL of hexane, vortexed and centrifuged at 3,000 rpm for 10 min. About 1.5 mL of the upper layer was collected and moved into 2 ml-GC vials. One μL of sample was injected into the column in the split mode (100:1). Fatty acid methyl esters were separated using a SP^TM^-2560 fused-silica capillary column (100 m × 0.25 mm, i.d. 0.20 μm film thickness; Supelco, Inc., USA) with a helium flow of 1.0 mL/min. The oven temperature was increased from 130 to 200 °C at 7 °C/min, held for 10 min, increased again to 250 °C at 5 °C/min, and finally held for 10 min. The temperatures of the injector and detector were 250 and 275 °C, respectively. Individual fatty acid was identified by comparison with the retention time and peak area of fatty acid standards (PUFA No. 2, 47015-U, Supelco, Inc., USA) and the proportion (%) was calculated by dividing its peak area with total peak area. Total fatty acid concentration (mg/100 g muscle) was calculated from meat crude fat content using a conversion factor of 0.92 for beef lipid and individual fatty acid concentration was calculated by multiplying its proportion with total fatty acids [14].

### Statistical analysis

Completely randomized block design was used. Data were analyzed using linear mixed model with the “lme4” package in R-version 3.3.2 (The R-foundation for Statistical Computing, Austria). Individual animal effects were included as a random term, while diet effects were used as a fixed term. Non-parametric data (carcass yield grade and quality traits) were analyzed using Kruskal-Wallis rank test. The statistical significance of the differences between means from different treatments was determined by Duncan’s multiple range test (*P* < 0.05) with “Agricolae” library.

## Results and discussion

Animals’ body weight gain and feed intake are shown in Table 2. No significant differences were found on final weight, total and daily weight gain of the experimental animals among diet groups and no acidosis-related incidence was reported during experiment. Average daily gain (kg) of RS, MH and TH groups were 0.89, 0.99 and 0.85, respectively. Although no statistical differences were found, MH-fed cattle tend to having higher weight gain than other groups. The differences ranged from 0.10 to 0.14 kg between MH-RS group and MH-TH group, respectively. Owens et al. [15] mentioned if acidosis does not occur in a group of feedlot cattle, then the supplementation of hay will increase feed intake but not weight gain. However, a lower intake of TH was observed as a result of its higher protein content than other diets. Diets also did not influence all carcass yield traits significantly (Table 3). However, MH group had significantly higher marbling score and carcass quality grade (grade 1 to 1+, *P* < 0.05) than the others (grade 2 to 1). Concentrate used in this study was categorized as relatively low protein and low energy concentrate based on its crude protein and total digestible nutrient levels. These attributes might contribute to carcass quality grade [16]. Beef from MH group had the highest crude fat content (*P* < 0.001) and the lowest moisture content (*P* < 0.01), which is in line with its higher marbling score. Marbling is important to determining juiciness, tenderness and overall eating satisfaction or palatability of the beef. Hanwoo is one of cattle breeds that can be able to produce highly marbled beef and this ability starts functioning from 12 months of age. The lipogenesis is accelerated with advancing maturity [17, 18]. Beside genetic factor, diet also influences marbling. Pethick et al. [19] mentioned that the net energy in diet can be enhanced by increasing the digestible fiber and lipid content of diet. The fat and NDF content of MH were relatively higher than those of RS and along with slightly higher intake than TH-fed group, it is assumed that MH group gained more available energy for fat deposition than the others. The differences on moisture and crude fat content in meat did not affect protein and ash content. Table 4 shows no differences were found on meat crude protein and ash content. These results are in line with Park et al. [20] who reported a negative correlation between fat and moisture content in beef. These suggest that supplementation of different hay products have no significant effects on animal performance and carcass yield, but MH in particular enhanced marbling and carcass quality grade by providing more available energy for fat deposition.

**Table 2:** Body weight gain and feed intake of Hanwoo steers fed with different supplementary hay

	RS	MH	TH	SEM	*P* value
Animal performance					
Initial weight (kg)	637.67	623.00	638.33	12.42	0.40
Final weight (kg)	771.00	772.33	765.17	14.57	0.39
Weight gain (kg)	133.33	149.33	126.83	5.28	0.21
Average daily gain (kg)	0.89	0.99	0.85	0.05	0.22
Daily dry matter intake					
Concentrate (kg)	10.26	10.09	9.53	-	-
Hay (kg)	0.99	1.01	0.72	-	-
Total (kg)	11.25	11.10	10.25	-	-
ADG/DDMI^a^	0.08	0.09	0.08	-	-

*RS* rice straw, *MH* mixed hay, *TH* timothy hay

*SEM* standard error of the means

Means within each row are not significantly different (*P* > 0.05)

^a^Average daily gain to total daily dry matter intake ratio

**Table 3:** Carcass traits of Hanwoo steers fed with different supplementary hay

	RS	MH	TH	SEM	*P* value
Yield traits					
Hot carcass weight (kg)	462.00	461.00	454.17	9.64	0.96
Backfat thickness (mm)	15.33	15.33	13.67	0.90	0.78
Ribeye area (cm^2^)	92.67	95.17	92.67	0.83	0.51
Yield index^c^	62.78	63.14	64.02	0.61	0.79
Yield grade^d^	2.50	2.33	2.17	0.32	0.73
Quality traits					
Marbling score^e^	3.17^b^	5.39^a^	3.67^b^	0.35	0.02
Meat color^f^	5.00	5.00	5.00	0.00	0.39
Fat color^g^	3.00	3.00	3.00	0.00	0.39
Firmness^h^	1.83	1.17	1.33	0.10	0.06
Maturity^i^	2.00	2.00	2.00	0.00	0.39
Quality grade^j^	2.50^b^	3.60^a^	2.67^b^	0.16	0.01

*RS* rice straw, *MH* mixed hay, *TH* timothy hay

SEM: standard error of the means

Different superscripts ^a~b^ in the same row indicate differences among diet groups (*P* < 0.05)

^c^65.834-[0.393 x Backfat thickness (mm)] + [0.088 x Ribeye area (cm^2^)]-[0.008 x Carcass weight (kg)] + 2.01

^d^A grade (yield index ≥69.00) = 3, B grade (66.00 ≤ yield index < 69.00) = 2, C grade (yield index <66.00) = 1

^e^Marbling score standard; No.1-No.7 (1 = devoid, 7 = abundant)

^f^ Meat color standard; No.1-No.7 (1 = brightly cherry red, 7 = extremely dark red)

^g^Fat color standard; No.1-No.7 (1 = white, 7 = dark yellow)

^h^Firmness score; 1–3 (1 = soft, 3 = firm)

^i^Maturity score; 1–3 (1 = youthful, 3 = mature)

^j^1^++^ grade = 5, 1^+^ grade = 4, 1 grade = 3, 2 grade = 2, 3 grade = 1

**Table 4:** Meat proximate composition

	RS	MH	TH	SEM	*P* value
Moisture (%)	67.51^a^	63.09^b^	64.84^ab^	0.65	<0.01
Crude fat (%)	10.99^c^	17.33^a^	14.96^b^	0.75	<0.001
Crude protein (%)	20.97	20.30	21.43	0.29	0.93
Ash (%)	0.89	0.88	0.87	0.01	0.33

*RS* rice straw, *MH* mixed hay, *TH*: timothy hay

*SEM* standard error of the means.

Different superscripts ^a~c^ in the same row indicate differences among diet groups (*P* < 0.05).

Color, which is the first characteristics noticed by consumers, plays an important role as an indicator of meat quality [21, 22]. Priolo et al. [23] found that iron from grass increases haemoglobin and myoglobin content in meat, whereas the yellowness is due to lipid-soluble β-carotene content from the cell wall of the grass. No significant differences on CIE L* value (lightness) of the meat among diet groups. Although no significant differences were found on the color of the carcass, the beef of MH samples had higher CIE a* value (redness) than the others and higher CIE b* value along with beef from RS group than that of TH group. These suggest that feeding MH supplies more iron than feeding RS and TH to Hanwoo steers.

Supplementation of MH tended to improve the deposition of fat in muscle. This high fat content affected the tenderness of the cooked samples, which agrees with Wood et al. [24] that total lipid content in muscle plays role in the tenderness. As shown in Table 5, beef from MH group, which contained 17.33% of fat, was more tender with lower shear force value and had overall softer texture with lower hardness, cohesiveness, gumminess, chewiness and resilience than the others. As no differences were found on meat pH among diet groups, no differences were found on cooking loss, drip loss and water holding capacity.

**Table 5:** Meat quality traits of Hanwoo steers fed with different supplementary hay

	RS	MH	TH	SEM	*P* value
pH_ultimate_	5.60	5.63	5.60	0.01	0.41
CIE L*	42.89	44.20	42.71	0.60	0.65
CIE a*	20.80^ab^	21.32^a^	18.82^b^	0.33	0.02
CIE b*	10.39^a^	10.83^a^	9.46^b^	0.18	<0.01
Drip loss (%)	6.55	6.68	5.09	0.31	0.16
Water-holding capacity (%)	55.36	56.79	56.10	1.08	0.13
Cooking loss (%)	31.25	31.57	30.82	0.22	0.24
Shear force (kg)	6.21^a^	4.40^b^	4.80^ab^	0.25	0.04
Hardness (kg)	7.44^a^	5.75^b^	6.26^ab^	0.23	0.03
Springiness (cm)	0.51	0.51	0.51	0.01	0.62
Cohesiveness	0.43^a^	0.36^b^	0.42^a^	0.01	0.02
Gumminess (kg)	3.22^a^	2.10^b^	2.62^ab^	0.13	0.02
Chewiness (kg.cm)	1.62^a^	1.06^b^	1.34^ab^	0.06	0.02
Resilience	0.25^a^	0.19^b^	0.22^a^	0.01	<0.01

*RS* rice straw, *MH*: mixed hay, *TH*: timothy hay

*SEM* standard error of the means

Different superscripts ^a~b^ in the same row indicate differences among diet groups (*P* < 0.05)

Ten fatty acids were identified in the beef samples and the concentration (mg/100 g muscle) was also calculated. Table 6 shows that differences were observed on the content of all identified fatty acids as the fat content of the beef from different diet groups was also different significantly. The concentration of total identified fatty acids was higher in MH-fed beef, followed by TH and RS-fed beef, respectively. The concentration of total saturated fatty acids (SFA) was higher in the beef of MH and TH groups than in RS-fed beef. Among diet groups, MH-fed beef contained higher concentration of myristic acid (C14:0), palmitic acid (C16:0) and stearic acid (C18:0), followed by TH-fed beef and RS-fed beef, respectively. MH-fed beef also contained higher concentration of monounsaturated fatty acids (MUFA) than the others including palmitoleic acid (C16:1n-7), oleic acid (C18:1n-9) and eicosenoic acid (C20:1n-9). Oleic acid was the major fatty acid, accounting for 46.76%, 47.19% and 47.09% for total fatty acids in the beef of RS, MH and TH group, respectively. These results agree with previous findings in Hanwoo beef [25]. No significant differences were found on the concentration of total polyunsaturated fatty acids (PUFA) between MH and TH-fed beef, while RS-fed beef had lower concentration than other diet groups. Among PUFA, the major contributor was linolenic acid (C18:2n-6), followed by alpha-linolenic acid (C18:3n-3), arachidonic acid (C20:4n6) and eicosapentaenoic acid (C20:5n3). Significant differences between MH and TH-fed beef were observed only on the concentration of alpha-linolenic acid (C18:3n-3). The concentration of alpha-linolenic acid (C18:3n-3) was higher in MH group, followed by TH and RS group, respectively. These differences led to lower the ratio of n-6 to n-3 fatty acids in the beef samples of MH group than in that of other diet groups. The inclusion of red clover (10% as fed) in MH could contribute in the deposition of n-3 fatty acids to muscle fat. Scollan et al. [26] reported that red clover supplementation can be used to manipulate fatty acid composition in beef, in particular to reduce ruminal biohydrogenation of PUFA. High activity of polyphenol oxidase in red clover [27] may provide protective effects against biohydrogenation of dietary alpha-linolenic acid (C18:3n-3) in rumen.

**Table 6:** Fatty acid concentration (mg/100 g muscle) of *longissimus thoracis* obtained from Hanwoo steers fed with different supplementary hay

Compound	RS	MH	TH	SEM	*P* value
C14:0	492^c^	727^a^	643^b^	38.75	<0.001
C16:0	2960^c^	4665^a^	4059^b^	226	<0.001
C16:1n-7	443^b^	740^a^	581^ab^	43.29	0.01
C18:0	992^c^	1551^a^	1342^b^	68.97	<0.001
C18:1n-9	4728^c^	7510^a^	6481^b^	259	<0.001
C18:2n-6	365^c^	528^a^	477^b^	14.25	<0.001
C18:3n-3	40.44^c^	92.46^a^	61.92^b^	3.58	<0.001
C20:1n-9	36.40^a^	54.20^a^	39.91^b^	2.87	<0.001
C20:4n-6	45.50^b^	63.77^a^	66.05^a^	4.51	<0.001
C20:5n-3	8.09^b^	12.75^a^	12.38^a^	0.86	<0.001
Total fatty acids	10111^c^	15944^b^	13763^a^	816	<0.001
∑SFA	4445^b^	6943^a^	6044^a^	312	<0.001
∑MUFA	5207^c^	8304^a^	7102^b^	285	<0.001
∑PUFA	459^b^	697^a^	618^a^	31.56	<0.001
∑n-6	410^b^	591^a^	544^a^	18.84	<0.001
∑n-3	48.53^c^	105^a^	74.31^b^	19.03	<0.001
n-6/n-3	8.46^a^	5.64^b^	7.31^a^	0.16	0.01

*RS* rice straw, *MH* mixed hay, *TH* timothy hay

*SEM* standard error of the means

*SFA* saturated fatty acids, *MUFA* monounsaturated fatty acids, *PUFA* polyunsaturated fatty acids

Different superscripts ^a~c^ in the same row indicate differences among diet groups (*P* < 0.05)

## Conclusions

Supplementation of RS, MH and TH during fattening to Hanwoo steers had no different effects on growth performance and carcass weight and yield grade. However, feeding MH, consisting 55% of orchard grass (*Dactylis glomerata* L.), 35% of tall fescue (*Festuca arundinacea*) and 10% of red clover (*Trifolium pratense*), resulted in higher quality grade, marbling score, meat fat content, redness and lower shear force, hardness and n-6 to n-3 fatty acids ratio. Based on these evidences, MH from local source could be used as supplementary hay for Hanwoo fattening program.

## Abbreviations

AOAC: The association of analytical communities
CIE: The Commission International De L’ecairage
MH: Mixed hay
MUFA: Monounsaturated fatty acids
PUFA: Polyunsaturated fatty acids
RS: Rice straw
SFA: Saturated fatty acids
TH: Timothy hay
TMR: Total mixed ration
WHC: Water holding capacity
